# Beyond overall accuracy: subgroup performance and threshold calibration of AI-assisted chest radiography for equity-oriented pulmonary tuberculosis screening

**DOI:** 10.3389/fmed.2026.1980048

**Published:** 2026-10-14

**Authors:** Wei Chen, Sixu Pan, Zhirong Zhang, Ying-ying Shen, Zhaokun Fan, Cheng-en Li

**Affiliations:** Department of Intensive Care Unit, The First Affiliated Hospital of Zhejiang Chinese Medical University (Zhejiang Provincial Hospital of Chinese Medicine), Hangzhou, China

**Keywords:** tuberculosis, artificial intelligence, chest radiography, computer-aided detection, threshold calibration, health equity

## Abstract

Artificial intelligence-assisted computer-aided detection (CAD) can expand access to chest radiography interpretation for pulmonary tuberculosis screening and triage, but high overall discrimination does not ensure safety at a deployed threshold. This focused narrative Mini Review synthesizes evidence on subgroup performance, threshold calibration, external validity, and implementation of CAD for adult pulmonary tuberculosis. Smear-negative, asymptomatic, and some HIV-associated disease may be less radiographically visible, increasing false-negative risk. Conversely, previous tuberculosis and competing chronic abnormalities reduce specificity and concentrate unnecessary confirmatory testing. Sex, age, geography, clinical setting, verification design, and software version can further alter operating performance. A numerical threshold is therefore not a portable algorithm property but a context-specific clinical resource-allocation policy. We propose a lifecycle in which programs define the intended use, lock the product and version, conduct representative local validation, select an operating point under explicit sensitivity and capacity constraints, audit clinically relevant subgroups, and monitor the complete care pathway. Stratified thresholds and multivariable models may improve selected settings, but current evidence does not justify replacing robust local calibration and governance. Prospective evaluations should compare threshold strategies under common verification standards and report missed cases, referral burden, treatment initiation, and patient-important outcomes.

## 1 Introduction

Chest radiography is a practical entry point for pulmonary TB screening and triage where expert readers and molecular testing are limited. AI-based computer-aided detection (CAD) can standardize chest x-ray interpretation and prioritize bacteriological confirmation. The World Health Organization (WHO) conditionally recommends CAD software as an alternative to human readers for interpreting digital chest radiographs for TB screening and triage among people aged 15 years or older, with low certainty of evidence, alongside local threshold calibration and ongoing monitoring (1). A 2025 WHO policy statement subsequently reported that six independently assessed CAD products met WHO performance standards and emphasized product-specific evaluation as brands and versions evolve (2). Neither document establishes a universal threshold. The clinical value of CAD also depends on infrastructure, workflow, testing, and follow-up (3).

Evidence has moved from algorithm development toward independent clinical evaluation. An early systematic review found that studies were mainly development-focused, often used same-source data, and rarely prespecified thresholds, making high area under the receiver operating characteristic curve (AUC) estimates inadequate proxies for deployment (4). Later individual participant data analyses and multicountry evaluations found strong aggregate discrimination, but fixed-score sensitivity and specificity varied across studies, countries, and products (5–7).

Once a continuous CAD score triggers action, the operating threshold determines who is missed and who undergoes confirmation. Performance can vary with HIV status, bacillary burden, previous TB, age, and sex; incomplete microbiological verification can distort apparent threshold performance, while software updates can shift score distributions despite small AUC changes (5, 8, 9). Fairness must therefore be assessed through the distribution of false-negative risk and unnecessary referral under a defined product version, population, reference standard, and resource constraint.

This focused narrative Mini Review examines adult pulmonary TB screening or triage with AI-assisted digital chest radiography. It addresses subgroup performance, threshold calibration, external validity, and implementation rather than algorithm architecture or new pooled estimates. We ask which groups experience clinically important performance shifts, when thresholds fail to transfer, and how local calibration, subgroup auditing, and lifecycle monitoring can support equity-oriented deployment.

Here, equity is not defined as equalized odds or identical error rates across groups. It is operationalized as whether a prespecified threshold produces clinically acceptable subgroup sensitivity, referral burden, and net benefit under explicit resource constraints. Minimum acceptable performance and trade-offs remain normative program decisions that should involve affected communities and implementers.

PubMed and Web of Science Core Collection were searched from inception to 15 August 2026 for pulmonary TB, chest radiography, and AI/CAD. Of 960 records, 666 remained after deduplication and 80 underwent detailed assessment; 26 informed the four core themes. Five policy or reporting-guideline sources provided recommendation context, and one targeted clinical source was added during revision to address radiographic overlap in people with HIV. Eligible reports addressed adult screening or triage, subgroup performance, calibration, external validation, implementation, or dataset shift. Pediatric, CT-led, drug-resistance, treatment-recommendation, and development-only studies without relevant external evaluation were excluded. One author conducted selection without duplicate review; no protocol was registered or formal risk-of-bias tool applied, and synthesis was narrative. Search strings, selection rules, and characteristics of the 26 core reports are in Supplementary File 1 and Supplementary Table S1.

## 2 Why overall accuracy is not enough

### 2.1 AUC and operating performance answer different questions

The AUC summarizes how well a CAD score ranks people with and without pulmonary TB across all possible thresholds. It is useful for comparing discrimination, but a screening program does not operate across the full receiver operating characteristic curve. It selects one threshold, which produces a particular sensitivity, specificity, referral proportion, and number of confirmatory tests. Consequently, similar AUCs can conceal clinically important differences at the intended operating point, while a modest AUC difference may matter little if two systems perform similarly at the required sensitivity (7, 10).

Scores are also product-specific rather than standardized probabilities. In a South African head-to-head evaluation of 12 commercial products, a score of 0.5 yielded sensitivities ranging from below 50% to 89.1%. At approximately 90% sensitivity, specificity ranged from 32.5% to 67.7% across products (11). These comparisons show why neither an AUC ranking nor a shared numerical cut-off can determine deployment performance. The relevant comparison is product- and version-specific performance at a clinically defined operating point in the intended population.

### 2.2 A threshold is a clinical resource-allocation policy

Selecting that operating point is not a purely statistical exercise. Lowering the threshold generally refers more people for molecular confirmation and reduces missed radiographically detectable TB; raising it reduces confirmatory testing but increases false negatives. Manufacturer defaults, a score of 0.5, and a Youden-index optimum each encode a trade-off, but none is inherently appropriate for a program's diagnostic capacity or tolerance for missed disease. Moreover, apparent threshold performance depends on who receives microbiological verification and how unverified TB status is handled (8).

The operational consequences are visible in a large case-finding project in the Philippines. Raising the qXR threshold from 0.5 to 0.68 increased the estimated proportion of molecular tests saved from 24.9% to 41.9%, while the proportion of missed cases increased from 4.4% to 10.0% (12). These are program-specific pseudo-estimates because most CAD-negative participants were not microbiologically tested; they illustrate the trade-off rather than prescribe a threshold. A defensible operating point should therefore be chosen for a defined use case, product version, population, reference standard, and confirmatory capacity, with uncertainty and subgroup effects reported alongside aggregate performance. Figure 1C summarizes the corresponding governance lifecycle.

**Figure 1:**
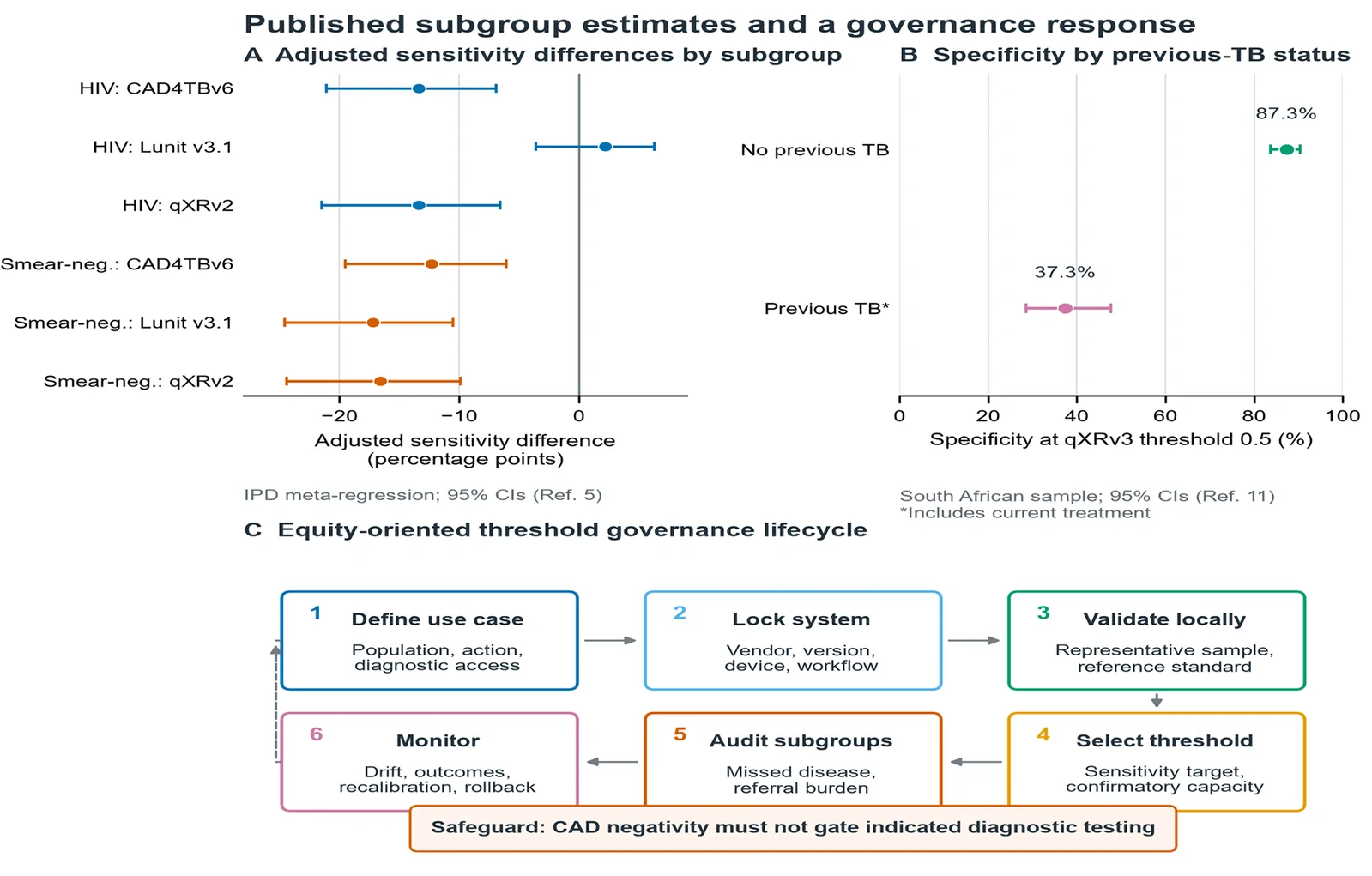
Published subgroup estimates and an equity-oriented governance response for AI-assisted chest radiography in pulmonary tuberculosis screening. **(A)** Adjusted absolute sensitivity differences from individual-participant-data meta-regression at meta-analysis-derived thresholds (CAD4TBv6, 54; Lunit INSIGHT v3.1.0.0, 16.68; qXRv2, 0.44). The comparisons are HIV-positive versus HIV-uninfected participants and smear-negative versus smear-positive TB. Negative values indicate lower sensitivity in the first named subgroup; points and bars show estimates and 95% confidence intervals (5). The source included symptomatic, self-referred adults from four studies; the qXR sensitivity model excluded sex because that model did not converge when sex was included. **(B)** qXRv3 specificity at a threshold of 0.5 among people with and without previous TB in a South African prevalence-survey-derived case-control sample; points and bars show estimates and 95% confidence intervals (11). The previous-TB category included participants receiving TB treatment at survey enrolment. **(C)** Six-stage lifecycle linking intended use, system locking, representative validation, threshold selection, subgroup audit, and monitoring. Panels A and B report separate studies and should not be pooled or interpreted as product rankings, score distributions, or portable threshold corrections. Panel C is an author-generated conceptual synthesis, not a prospectively validated implementation protocol. No patient images or reconstructed score distributions are shown.

## 3 Who is disadvantaged at a fixed threshold?

The recurrent pathways through which a fixed threshold produces missed disease or excess referral, together with minimum deployment responses, are summarized in Table 1. Figures 1A,B visualizes two documented patterns: subgroup differences in sensitivity and lower specificity among people with previous TB.

**Table 1:** Recurrent failure modes, affected groups, and deployment responses.

Failure mode	Typical groups or settings	Consequence at a fixed threshold	Minimum deployment response	References
Reduced radiographic visibility	HIV or advanced HIV; smear-negative, low-bacillary-load, asymptomatic or subclinical TB	Disproportionate false negatives and unsafe rule-out	Do not use CAD negativity to withhold or delay indicated diagnostic testing; validate any threshold change prospectively	(5, 13–16)
Residual or competing abnormalities	Previous TB; older adults; miners or silicosis; high-abnormality inpatient settings	False positives, excess confirmation, and concentrated referral burden	Audit separately; consider prior-image review, sequential assessment, or validated stratification	(11, 17, 18, 22)
Geographic and spectrum shift	Countries and sites; community screening versus symptomatic triage or inpatient care	Both error types; failure of a previously acceptable operating point	Perform representative local validation and calibration for the intended use case	(6, 7, 21)
Reference-standard and verification bias	CAD-triggered sputum testing; expert-CXR versus microbiological targets; incomplete negative verification	Biased apparent accuracy, threshold estimates, and product ranking	During validation, verify all participants or use probability-based sampling of negatives with appropriate weighting; define the target condition explicitly	(8, 12, 23, 24)
Product and version drift	Different vendors, score scales, devices, or updated software	Silent change in referral volume, specificity, and missed cases	Lock the version; use shadow testing, recalibration, capacity checks, and a rollback plan	(9, 25)
Resource-constrained thresholding	Limited molecular-test capacity; incomplete sputum submission; loss to follow-up	Explicit trade-off between tests saved and cases missed	Report tests saved, cases missed, sputum completion, treatment initiation, and downstream outcomes together	(12, 27, 29)

AUC, area under the receiver operating characteristic curve; CAD, computer-aided detection; CXR, chest x-ray; TB, tuberculosis. Deployment responses are author synthesis and have not been validated as a complete implementation protocol.

### 3.1 Disproportionate false negatives: low-visibility and high-risk disease

HIV-associated TB is a safety concern because advanced immunosuppression can produce less typical radiographic abnormalities. In an individual participant data meta-analysis, HIV-positive status was associated with adjusted 13.4-percentage-point sensitivity reductions for CAD4TBv6 and qXRv2 at meta-analysis-derived thresholds; the Lunit estimate was 2.2 points higher and its confidence interval included no difference (5) (Figure 1A). These model-based contrasts are not portable correction factors. A prospective Zambian validation found 90% sensitivity and 72% specificity among HIV-negative participants but 82% and 65% among those with HIV at a prespecified threshold (13). In an advanced-HIV clinic cohort, qXR identified 58 of 116 recorded TB diagnoses at threshold 0.5, but only 24 diagnoses were bacteriologically confirmed, precluding an unbiased accuracy estimate (14). CXR findings can also be etiologically nonspecific: in a single-centre study of smear-negative, HIV-positive patients with pneumonia-like syndromes, TB, bacterial pneumonia, and Pneumocystis pneumonia overlapped radiographically, with no uniquely distinguishing CXR feature (16). Thus, CXR-CAD must not substitute for indicated diagnostic testing; a negative score must not prevent or delay microbiological investigation. Radiographic overlap is a modality-level limitation; better training representation alone cannot guarantee reliable etiological discrimination (13–16).

Low bacillary burden creates a related false-negative pattern. At the meta-analysis-derived thresholds in Figure 1A, adjusted sensitivity was 12.3–17.2 percentage points lower for smear-negative than smear-positive TB (5). Community screening also shifts the spectrum toward asymptomatic disease. In rural South Africa, almost 80% of confirmed cases were asymptomatic and 19 of 99 had radiographs read as normal by expert radiologists. Microbiologically confirmed TB may therefore be radiographically subtle or judged normal, limiting reliance on CXR-based triage to exclude disease (15). Lowering the threshold may recover some subtle disease but increases referrals.

Women have shown lower sensitivity at selected operating points in several datasets. For example, CAD4TB sensitivity was 88% in women and 98% in men in a prospective culture-referenced study (10). Multicountry analyses have repeated this signal, but its magnitude and direction are not uniform across products and settings (6, 7). Differences in disease burden, care-seeking, image appearance, and correlated clinical characteristics may all contribute. Sex should therefore be treated as a prespecified audit dimension, not as a sufficient causal explanation or automatic justification for a separate threshold.

### 3.2 Disproportionate false positives: residual and competing abnormalities

Previous TB provides the most consistent evidence of disproportionate over-referral. At operating points near 90% sensitivity, two CAD systems achieved approximately 60% specificity among people without previous TB but only 22%–24% among those with previous TB (17). A South African product comparison found a similar pattern: qXR specificity at a score of 0.5 was 87.3% (95% CI, 83.7–90.4) without previous TB and 37.3% (28.5–47.7) with previous TB (11) (Figure 1B). Residual fibrosis, volume loss, and other post-TB abnormalities can remain visually compatible with active disease. Raising the threshold may reduce referrals but can also miss recurrent TB; a distinct follow-up pathway using prior images, clinical history, or sequential review may therefore be more defensible than threshold adjustment alone.

Older age, male sex, low body mass index, silicosis, and other chronic abnormalities can also shift score distributions and reduce specificity, although these factors frequently overlap. An ex-miner study showed that high AUC did not ensure adequate specificity at a high-sensitivity operating point in a population with substantial silicosis and previous TB (18). Diabetes illustrates the danger of overgeneralizing from small special-population studies: in one cohort, increasing the CAD4TB threshold from 50 to 70 reduced Xpert use but missed 20 of 74 TB cases (19). These results support targeted validation in high-risk populations, not the assumption that a diagnostic label alone determines an optimal threshold.

### 3.3 Intersectionality and geography

Subgroup attributes do not act independently. Age, sex, HIV, previous TB, symptoms, occupation, and country jointly shape disease visibility and the distribution of non-TB abnormalities. In a seven-country head-to-head study, a common threshold produced lower sensitivity in women and people with HIV, but lower specificity in men, older adults, and people with previous TB; performance also varied substantially by country (6). One-variable subgroup tables can therefore conceal relevant intersections and site effects. Yet greater stratification is not automatically better: in a Ugandan community dataset, age-sex-specific thresholds improved sensitivity by only 1.9 percentage points at matched specificity (20). These disparities are context-dependent signals requiring audit rather than proof of a single causal mechanism.

## 4 Why thresholds fail to travel

### 4.1 Spectrum and domain shift

A threshold calibrated in a symptomatic clinic encounters a different score distribution in community screening, hospitals, prisons, mines, or migrant programs. Differences in symptom status, bacillary burden, previous lung disease, prevalence, acquisition hardware, and image processing can alter both true- and false-positive scores. In an early dataset-shift study, AUC fell from 0.85 on a same-source test set to 0.71 on an external dataset (21). In hospitalized patients in Lima, qXR retained approximately 91% sensitivity but only 32%–44% specificity, consistent with a setting rich in competing pulmonary abnormalities (22). Threshold transport is therefore a spectrum and domain problem even when the software is unchanged.

Transport failure is not inevitable. In a prospective five-country symptomatic cohort, CAD4TB achieved 90% sensitivity and 70.3% specificity at a cohort-optimized cut-off (7). This shows that a product can meet target performance across multiple sites under harmonized eligibility and verification, but not that the same numerical cut-off transfers unchanged. The evidence supports testing transportability rather than assuming that every threshold will fail.

### 4.2 Reference-standard and verification bias

Apparent threshold failure can also arise from evaluation design. In a Lesotho prevalence survey, alternative approaches to unverified TB status changed estimated prevalence from 0.83% to 2.72% and materially altered apparent operating performance (8). The target condition matters as well: in a migrant study, Lunit ranked highest against microbiological TB, whereas CAD4TB ranked highest against expert radiographic abnormality (23). These are different clinical questions, not contradictory rankings.

Selective verification is especially consequential when sputum testing is triggered by symptoms or a positive CAD result. Among household contacts, routine single-sputum Xpert detected only 7 of 23 baseline TB cases, and more than 30% of people with a high CAD score but an initially negative routine test were later classified as TB after intensified sampling or follow-up (24). Conversely, programs that rarely test CAD-negative participants cannot establish the safety of a negative result, as illustrated by the Philippines implementation study (12). All-participant verification, probability-based verification of CAD-negative participants with appropriate weighting, or other bias-adjusted analysis is therefore integral to threshold validation rather than a secondary statistical detail.

### 4.3 Product and version drift

Numerical scores are not interchangeable across vendors or stable across updates. Earlier comparisons showed that new CAD4TB and qXR versions substantially improved AUC and performance at high-sensitivity operating points, but did not eliminate poor performance among people with previous TB (25). More importantly, an update can move the score scale without improving the operating point that matters. In a Lunit version comparison, the median score rose from 0.35 to 0.61; carrying forward the old threshold reduced specificity from 45.8% to 23.1%, whereas recalibration restored 90% sensitivity without a significant specificity advantage over the older version (9). A software update should thus be governed as a change to a clinical intervention, with version locking, shadow evaluation, recalibration, and a rollback plan.

## 5 From calibration to equity-oriented deployment governance

### 5.1 Define the use case before selecting a threshold

Threshold selection should begin with the decision pathway. Programs must specify CAD's use, eligibility, minimum sensitivity, confirmatory capacity, and action after a negative result. Local validation should use the intended software version and acquisition system in a representative sample. The target condition and reference standard must be explicit; validation should verify all participants or sample CAD-negative participants probabilistically with appropriate adjustment. Thresholds and key subgroup analyses should be prespecified to avoid treating a locally optimized result as validated performance (6, 8). Figure 1C links these requirements in a six-stage lifecycle.

Because score scales and default cut-offs are vendor-controlled, evaluations should disclose manufacturer involvement, funding, data access, and who selected the operating point. Independent, version-specific validation is particularly important when developers contribute to study design or analysis (2, 31, 32).

### 5.2 Choose the least complex strategy that improves net benefit

A locally calibrated universal threshold is simplest to communicate and monitor, but does not remove subgroup disparities. Stratified thresholds may help when score distributions differ predictably, yet gains can be modest and rules difficult to govern. The Ugandan age-sex analysis improved sensitivity from 75.0% to 76.9% at the same specificity, a statistically detectable but operationally small gain (20). Multivariable models offer another route: combining continuous CAD scores with age, sex, HIV, and previous TB increased specificity by about five percentage points at 90% sensitivity in a retrospective individual-level analysis (26). This remains proof-of-concept evidence until the model, calibration, and action threshold are locked and externally tested.

For people with HIV at high risk of TB, access to diagnostic testing should be independent of CAD positivity. For adults and adolescents with HIV who have TB signs or symptoms, screen positive for TB, are seriously ill, or have advanced HIV disease, WHO recommends concurrent respiratory low-complexity automated nucleic acid amplification testing and urine lateral flow lipoarabinomannan testing as the initial diagnostic strategy (28). Persistent suspicion warrants further clinical and microbiological evaluation as indicated. CAD can remain an adjunct for screening or triage but should not gate this diagnostic pathway (14, 28).

### 5.3 Monitor the full decision pathway

As summarized in Figure 1C, the proposed governance framework continues after launch. Before deployment, programs should record the vendor, version, device, threshold, reference standard, expected referral volume, and subgroup audit plan. Monitoring should cover subgroup sensitivity and specificity where verification permits, the proportion referred, inability to provide a sputum specimen, laboratory turnaround, loss to follow-up, and treatment initiation. A Vietnamese implementation study showed that human-CAD agreement and apparent threshold performance changed over time alongside training, referral, and program processes (29). These are properties of the sociotechnical pathway, not only of the algorithm.

Updates ideally require shadow running against the previous version, recalibration, laboratory-capacity stress testing, documented approval, and a rollback option (9). These measures are not equally feasible everywhere. Because throughput and pricing materially affect affordability (30), a proposed minimum resource-constrained package may prioritize version logs, referral-volume monitoring, periodic probability-based verification with appropriate analysis, and predefined escalation triggers. Overall AUC or positivity rates alone can miss offsetting subgroup harms.

## 6 Discussion, limitations, and research priorities

### 6.1 Evidence synthesis and implications

As synthesized in Table 1, the evidence is most consistent for two opposing harms. Previous TB and competing chronic abnormalities reduce specificity, concentrating confirmatory testing among people with residual radiographic changes. Conversely, smear-negative, asymptomatic, and some HIV-associated disease is less radiographically visible, increasing false-negative risk. Sex, age, and geographic differences recur, but heterogeneous populations, correlated characteristics, and *post hoc* analyses prevent simple causal attribution (5, 6, 17). Fairness is therefore not whether subgroup AUCs are identical, but whether a defined threshold allocates missed disease and confirmatory burden acceptably across the intended population.

### 6.2 Limitations of the evidence and this review

This is a focused narrative Mini Review, not a systematic review or meta-analysis. Estimates were not pooled because settings, reference standards, verification, products, versions, and thresholds were not exchangeable; percentage-point differences are illustrative, not directly comparable. One author selected evidence without duplicate review or formal risk-of-bias scoring, so selection bias remains possible. Many subgroup analyses are underpowered or *post hoc*, patient-important outcomes were rarely reported, and economic evidence was largely modelled (30). Rapid updates make older estimates time-sensitive (9). The source added during revision supports only a narrow observation about radiographic overlap in smear-negative people with HIV, not all immunocompromised populations or screening settings (16).

### 6.3 Research priorities

Future evaluations should lock versions and thresholds in representative multicountry cohorts. During validation, all participants should receive microbiological verification or CAD-negative participants should be sampled using a known probability scheme with appropriate weighting or bias adjustment (8). Protocols should prespecify intersectional subgroups and report calibration, uncertainty, referral volume, missed cases, net benefit, and downstream outcomes. Diagnostic-accuracy studies should follow the final STARD-AI reporting guideline, while imaging-focused evaluations should also consider the CLAIM 2024 Update. Together, these guidelines emphasize intended use, data provenance, reference standards, external testing, uncertainty, subgroup performance, human-AI interaction, and funding transparency (31, 32). Threshold strategies should be compared under common population and capacity constraints (20, 26), and studies should assess treatment initiation and harmful delay, not only retrospective accuracy.

### 6.4 Conclusion

Overall AUC is an incomplete basis for deploying AI-assisted chest radiography in TB screening. A threshold is a context-specific policy that distributes missed disease and confirmatory resources. Current evidence supports prespecified local validation, appropriately analyzed verification of CAD-negative participants, subgroup auditing, and recalibration after software updates. CAD should remain an adjunct and must not gate indicated diagnostic testing in people with HIV or other immunocompromised patients. Personalized strategies are promising but require prospective evidence of superior net benefit and acceptable implementation burden.
