# Supplementary material for: Beyond overall accuracy: subgroup performance and threshold calibration of AI-assisted chest radiography for equity-oriented pulmonary tuberculosis screening
Source: Front Med (Lausanne). 2026 Oct 14;13:1980048. doi: 10.3389/fmed.2026.1980048 (PMC13714600; doi:10.3389/fmed.2026.1980048)
Supplement: Supplementary file 1 [file Table1.docx]

Supplementary File 1. Search strategy and narrative evidence-selection process

# Review design

This was a focused narrative Mini Review. The search was designed to make evidence identification transparent, but the review was not preregistered, screening was not performed independently in duplicate, and no formal study-level risk-of-bias instrument was applied. The workflow should therefore not be interpreted as a systematic review.

# Information sources and date

PubMed and Web of Science Core Collection were searched from database inception to 15 August 2026. Backward and forward citation checking was used for directly relevant reviews, validation studies, and WHO policy documents.

# PubMed search

```
(
  "Tuberculosis, Pulmonary"[MeSH Terms] OR "pulmonary tuberculosis"[Title/Abstract] OR tuberculosis[Title/Abstract]
) AND (
  "Radiography, Thoracic"[MeSH Terms] OR "chest radiograph"[Title/Abstract] OR "chest radiographs"[Title/Abstract]
  OR "chest x-ray"[Title/Abstract] OR "chest x-rays"[Title/Abstract] OR CXR[Title/Abstract]
  OR "digital radiography"[Title/Abstract]
) AND (
  "Artificial Intelligence"[MeSH Terms] OR "artificial intelligence"[Title/Abstract]
  OR "machine learning"[Title/Abstract] OR "deep learning"[Title/Abstract]
  OR "computer-aided detection"[Title/Abstract] OR "computer aided detection"[Title/Abstract]
  OR "computer-assisted detection"[Title/Abstract] OR "computer assisted detection"[Title/Abstract]
  OR CAD4TB[Title/Abstract] OR qXR[Title/Abstract] OR "Lunit INSIGHT"[Title/Abstract] OR InferRead[Title/Abstract]
)
```

# Web of Science Core Collection search

TS=(("pulmonary tuberculosis" OR tuberculosis)
AND ("chest radiograph*" OR "chest x-ray*" OR CXR OR "digital radiograph*")
AND ("artificial intelligence" OR "machine learning" OR "deep learning"
OR "computer-aided detection" OR "computer aided detection"
OR "computer-assisted detection" OR "computer assisted detection"
OR CAD4TB OR qXR OR "Lunit INSIGHT" OR InferRead))

# Focused prioritization terms

Subgroup, stratified, fairness, equity, bias, generalizability, transportability, external validation, calibration, threshold, implementation, deployment, older age, HIV, previous tuberculosis, sensitivity, and specificity were used to prioritize detailed reading. They were not mandatory in the primary search.

# Eligibility criteria

Eligible reports addressed adults undergoing pulmonary TB screening or triage using digital chest radiography interpreted by AI/CAD and contributed evidence on operating thresholds, subgroup performance, external validation, verification, software-version changes, or implementation. Methodologically informative external-dataset studies were eligible when they directly examined transportability or dataset shift. Pediatric, CT-led, drug-resistance, treatment-recommendation, non-pulmonary, development-only, and dataset-competition reports were excluded from the core synthesis. One author conducted screening and evidence selection; independent duplicate screening and a formal risk-of-bias assessment were not performed.

# Record flow

PubMed: 452; Web of Science: 508; total before deduplication: 960; after deduplication: 666; retained for detailed assessment: 80. Twenty-six reports directly informed the review's four organizing themes. The other 54 detailed-assessment records either did not contribute directly to those themes or met the stated exclusions. The final manuscript cited 30 sources: 26 core evidence reports and four policy or reporting-guideline sources used for recommendations and reporting context.

# Synthesis approach

Evidence was synthesized narratively because studies differed in setting, eligibility, reference standard, verification, product, software version, threshold selection, and subgroup reporting. Numerical differences were treated as study-specific examples, not exchangeable effect estimates.

# Supplementary Table S1. Characteristics and interpretive role of the 26 core evidence reports

This table supports transparent narrative synthesis; it is not a formal risk-of-bias assessment. Counts and reference-standard descriptions are reported at the level needed to interpret each source's role in this review. CAD, computer-aided detection; CXR, chest radiography; ILO, International Labour Organization; MGIT, mycobacteria growth indicator tube; TB, tuberculosis.

| **Ref.** | **Study design and setting** | **Population** | **AI/CAD system** | **Reference standard and verification** | **Role in this review** | **Principal limitation for interpretation** |
| --- | --- | --- | --- | --- | --- | --- |
| 3 | Narrative implementation review; low- and middle-income TB programs | Program reports and diagnostic studies across heterogeneous screening pathways | Multiple AI/mobile CXR systems | Varied across included sources; no unified reference standard | Implementation context and operational constraints | Narrative synthesis; not a source of pooled diagnostic effect estimates |
| 4 | Systematic review; 53 articles (40 development, 13 clinical) | Adults represented in early CAD development and clinical evaluation studies | Multiple systems, with clinical evidence concentrated on CAD4TB | Microbiological and/or human-reader standards varied by study | Historical evidence base and sources of bias | Older products and heterogeneous designs limit current quantitative relevance |
| 5 | Individual-participant-data meta-analysis; Pakistan, South Africa, Tanzania, and Zambia | 3,727 symptomatic adults; 645 microbiologically positive; 17% living with HIV | CAD4TB v6, Lunit INSIGHT CXR v3.1, qXR v2 | At least one culture or Xpert result | Cross-setting and subgroup performance | Older software versions and residual between-setting heterogeneity |
| 6 | Prospective multinational head-to-head evaluation; seven countries | 3,901 adults with cough for at least 2 weeks | CAD4TB, qXR, Lunit, DrAid, Genki, InferRead, and Radify | Stringent composite based on Xpert Ultra and culture | Same-CXR product comparison and transportability | Symptomatic spectrum and offline evaluation; some culture-based exclusions |
| 7 | Prospective multinational diagnostic study | 1,392 adults with persistent cough in five countries | CAD4TB v7 combined with host-response or C-reactive-protein tests | Two to three sputum specimens tested by Xpert Ultra and culture | Sequential triage algorithms and subgroup instability | Thresholds optimized within the cohort; country and sex performance varied |
| 8 | National TB prevalence survey; Lesotho | 17,070 adults; 5,214 with known TB status; 142 TB cases | CAD4TB v6 and v7 | Xpert Ultra plus liquid culture, with strategies for unverified participants | Population screening and consequences of incomplete verification | Verification assumptions materially influence estimates |
| 9 | Programmatic paired-version analysis | 2,708 high-risk individuals with valid Xpert; 280 positive; enriched for older age and previous TB | Lunit INSIGHT CXR v3.1 versus v3.9 | Single Xpert result after selective testing | Software-version drift | Selected image library and selective testing limit generalizability |
| 10 | Prospective diagnostic study; Karachi, Pakistan | 2,198 symptomatic adults; 272 culture-positive; 99.5% HIV-negative | qXR v2 and CAD4TB v6 | Two sputum cultures | Product comparison and subgroup performance | Single setting; CAD4TB operating point was data-derived |
| 11 | Enriched evaluation nested in a South African prevalence survey | 774 adults (258 positive and 516 negative) | Twelve commercial CAD products | Xpert Ultra or MGIT culture | Broad same-CXR product comparison | Enrichment and partial verification reduce direct programmatic transportability |
| 12 | Large programmatic observational study; Philippines | 52,840 screened; 47,817 received CXR; 5,740 had qXR plus molecular testing; 749 positive | qXR v3 | Molecular testing triggered by CAD score, symptoms, or human reading | Illustration of verification-workflow bias | Severe selective verification; reported miss fractions are program-specific pseudo-estimates |
| 13 | Prospective offline validation; three sites in Zambia | 1,827 adults; 192 TB cases; 35.5% living with HIV | Google TB AI plus an abnormality-detection model; prespecified thresholds | Culture or Xpert Ultra | Prospective external validation and prespecified operating points | Offline, single-city evaluation with developer involvement |
| 14 | Retrospective analysis; advanced-HIV cohort in Maputo, Mozambique | 487 enrolled; 238 with CXR; 116 recorded TB diagnoses | qXR v4 at threshold 0.5 | Recorded clinical or bacteriological diagnosis; only about one fifth bacteriologically confirmed | High-risk subgroup and pathway constraints | Substantial reference-standard and missing-data bias |
| 15 | Community-based cross-sectional study; rural South Africa | 9,914 adults; 99 microbiologically positive; 79.8% of cases asymptomatic | CAD4TB v5 prospectively and v6 retrospectively | Xpert plus culture after selective sputum collection | Low-symptom-spectrum performance | Incomplete verification and software-version change |
| 16 | Cross-sectional symptomatic evaluation | 1,884 adults; 452 with previous TB | CAD4TB v7 and qXR v3 | Xpert | Prior-TB subgroup performance and threshold choice | Xpert-only standard and post hoc subgroup thresholds |
| 17 | Field comparison; rural South African former miners | 501 consecutive chest radiographs | CAD4TB v7, CAD4Silicosis v1.0, and XrayAME v1 | Occupational-medicine specialist readings using ILO methods; no microbiology | Comorbidity, scarring, and reference-standard mismatch | Cannot distinguish active from previous TB and lacks bacteriological confirmation |
| 18 | Cross-sectional diagnostic study; Karachi, Pakistan | 694 adults with diabetes; 74 Xpert-positive | CAD4TB v3.07 | Contemporaneous Xpert for all participants | Diabetes subgroup and threshold-performance trade-offs | Small, single-setting study using an Xpert-only standard |
| 19 | Community screening study; Uganda | 52,835 screened; 7,219 with valid Xpert among those with qXR score at least 0.1; 382 positive | qXR v3.2 | Xpert above the programmatic CAD threshold; outcomes below threshold modelled | Age- and sex-stratified threshold implications | Selective verification and modelling assumptions |
| 20 | External-dataset dataset-shift experiment; China and United States | Shenzhen dataset (662) and NIH dataset (10,000) | Inception-v3 classifier | Bacteriological/clinical labels in Shenzhen versus mined non-specific labels in NIH | Mechanistic example of dataset and label shift | Not a clinical external-validation study; reference labels are not comparable |
| 21 | Prospective hospital-based triage and screening study; Lima, Peru | Triage cohort 419 (387 cultured; 65 culture-positive); screening cohort 184 | qXR v3 and v4 | Culture as the principal standard | Clinical pathway and version comparison | Screening arm had too few confirmed events for stable estimates |
| 22 | International multicenter migrant evaluation | 1,769 in microbiological analysis and 2,812 in radiographic analysis | Lunit v4.9, qXR v2, and CAD4TB v6 | Up to three cultures with or without Xpert; expert CXR for radiographic TB | Cross-country and spectrum transportability | Referral enrichment and selective verification |
| 23 | Prospective household-contact cohort | 483 adult contacts; median follow-up 4.6 years; 23 prevalent and 38 incident cases | CAD4TB v7, qXR v3, and Lunit v3.1 | Enhanced two- to three-specimen Xpert/culture plus longitudinal follow-up | Risk prediction beyond prevalent disease | Few outcome events and context-specific follow-up |
| 24 | Paired software-version comparison using an enriched multinational image set | 12,890 chest radiographs; 4,308 Xpert-positive | CAD4TB v6/v7 and qXR v2/v3 | Xpert | Version-dependent score and threshold changes | Enriched shared dataset; no direct program outcomes |
| 25 | Retrospective individual-participant-data prediction modelling | 4,733 complete cases from 5,640 participants; 805 microbiologically positive | CAD4TB v7 or qXR v3 plus demographic and clinical variables | Original-study culture and/or Xpert | Context-aware threshold models | Proof of concept with bootstrap internal validation only |
| 26 | Prospective registered diagnostic study; Lesotho and South Africa | 1,392 symptomatic adults; 48.3% living with HIV | CAD4TB v7 combined with C-reactive protein or expert reading | Composite culture or Xpert Ultra | Composite triage pathways | Threshold selection and evaluation occurred within the same study |
| 27 | District-level implementation study; Vietnam | 24,945 chest radiographs; 2,061 participants with CXR and valid Xpert | qXR v3 at threshold 0.60 | Selective Xpert testing | Monitoring, implementation, and human-CAD workflow | Incomplete verification; not a full diagnostic-accuracy estimate |
| 28 | Economic scenario model; Pakistan | Modelled screening strategies rather than an enrolled clinical cohort | CAD4TB, InferRead, Lunit, and qXR versus radiologist reading | Vendor pricing, staffing, training, and strategy assumptions | Cost and feasibility of alternative workflows | Modelled costs without observed clinical or equity outcomes |
